# Clinical benefits and risks of the wall-push technique in endovascular repair of abdominal aortic aneurysm with severely angulated neck: a case report

**DOI:** 10.1093/ehjcr/ytag555

**Published:** 2026-08-05

**Authors:** Xinqiang Ma, Weiguo Fu, Lixin Wang

**Affiliations:** Department of Vascular Surgery, Zhongshan Hospital, Fudan University, 180 Fenglin Road, Shanghai 200032, China; Department of Vascular Surgery, Vascular Surgery Institute of Fudan University, 180 Fenglin Road, Shanghai 200032, China; National Clinical Research Center for Interventional Medicine Zhongshan Hospital, Fudan University, 180 Fenglin Road, Shanghai 200032, China; Department of Vascular Surgery, Zhongshan Hospital, Fudan University, 180 Fenglin Road, Shanghai 200032, China; Department of Vascular Surgery, Vascular Surgery Institute of Fudan University, 180 Fenglin Road, Shanghai 200032, China; National Clinical Research Center for Interventional Medicine Zhongshan Hospital, Fudan University, 180 Fenglin Road, Shanghai 200032, China; Key Laboratory of Panvascular Disease Precision Medicine, Zhongshan Hospital Xiamen, Fudan University, 668 Jinhu Road, Xiamen 361015, China; Department of Vascular Surgery, Zhongshan Hospital, Fudan University, 180 Fenglin Road, Shanghai 200032, China; Department of Vascular Surgery, Vascular Surgery Institute of Fudan University, 180 Fenglin Road, Shanghai 200032, China; National Clinical Research Center for Interventional Medicine Zhongshan Hospital, Fudan University, 180 Fenglin Road, Shanghai 200032, China; Key Laboratory of Panvascular Disease Precision Medicine, Zhongshan Hospital Xiamen, Fudan University, 668 Jinhu Road, Xiamen 361015, China

**Keywords:** Iatrogenic aortic dissection, Abdominal aortic aneurysm, Tortuous neck, EVAR, Wall-push technique, Case report

## Abstract

**Background:**

Endovascular aneurysm repair (EVAR) has become the preferred treatment for infrarenal abdominal aortic aneurysms (AAA), offering reduced morbidity and faster recovery compared with open surgery. However, patients with complex neck anatomy pose significant technical challenges, and current treatment approaches carry inherent risks. Learning from and summarizing experiences from these complex cases is crucial for advancing EVAR techniques.

**Case summary:**

An 80-year-old female with a 10-year history of conservatively managed infrarenal AAA complained of intermittent abdominal pain for 1 week. Computed tomography angiogram (CTA) demonstrated a tortuous infrarenal AAA with a tapered neck. Aneurysmal dilatation and severe calcification of bilateral iliac arteries and tortuous aortoiliac access were also shown. EVAR was performed successfully using Gore Excluder C3 stent-grafts (W.L. Gore & Associates, Flagstaff, AZ, USA). During deployment, inadvertent forward displacement of the guidewire caused an iatrogenic abdominal aortic dissection above the left renal artery. The false lumen was immediately coil-embolized with five interlock coils, successfully excluding the dissection flap and restoring antegrade flow. The procedure was completed uneventfully, with the stent-graft well positioned, patent, and free of endoleak.

**Discussion:**

The case highlights both the utility and risks of advanced EVAR techniques in complex anatomy. The wall-push technique, while useful for severe neck angulation, requires meticulous guidewire control and carries risk of vessel injury. Key preventive measures include continuous fluoroscopic monitoring, team communication, consideration of safety wires, and careful preoperative assessment. The learning curve for advanced techniques is steep, emphasizing the need for proper training, experience, and comprehensive preoperative planning using three-dimensional CTA reconstruction.

Learning points

- The wall-push technique effectively navigates severely angulated aortic necks but requires meticulous guidewire control to prevent iatrogenic dissection.
- Iatrogenic aortic dissection during endovascular aneurysm repair can be successfully managed with prompt coil embolization of the false lumen.

## Introduction

Endovascular aneurysm repair (EVAR) has become the preferred treatment for infrarenal abdominal aortic aneurysms (AAA), offering reduced morbidity and faster recovery compared with open surgery. However, patients with complex neck anatomy pose significant technical challenges. Here, we present a case of an infrarenal AAA with a severely angulated neck, complicated by iatrogenic dissection during the wall-push technique.

## Summary figure

**Figure:**
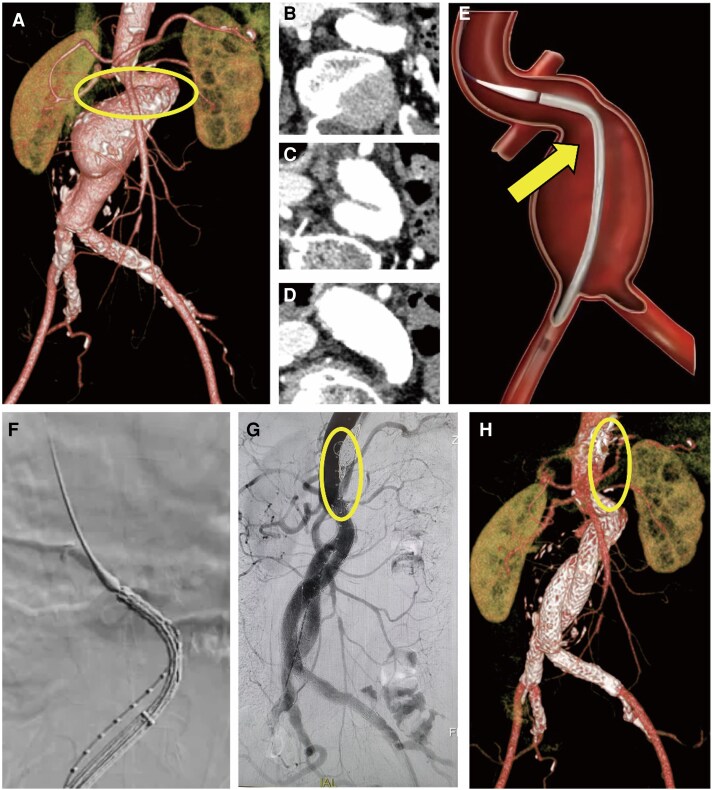


## Case presentation

The chronological course of the patient’s clinical care, from initial AAA diagnosis through the planned surveillance schedule, is summarized in *Table 1*.

**Table 1:** Timeline of key events

Time	Event
10 years prior to admission	Initial diagnosis of infrarenal AAA Conservative management with annual surveillance
2 months prior to admission	Onset of persistent abdominal pain
Day 0 (Admission)	CTA performed showing 5.8 × 5.5 cm AAA with 75° infrarenal neck angulation. (shown in *Figure 1A–1D*)
Day 2	EVAR performed; iatrogenic dissection occurred during wall-push technique; managed with coil embolization (5 Interlock coils); successful aneurysm exclusion. (shown in *Figure 1F–1G*)
1-month follow-up	Asymptomatic; stable sac diameter; thrombosed false lumen; patent renal arteries (shown in *Figure 1H*)
6 and 12 months (planned)	Scheduled surveillance CTA.

AAA, abdominal aortic aneurysms; CTA, computed tomography angiography; EVAR, endovascular aneurysm repair.

**Figure 1:**
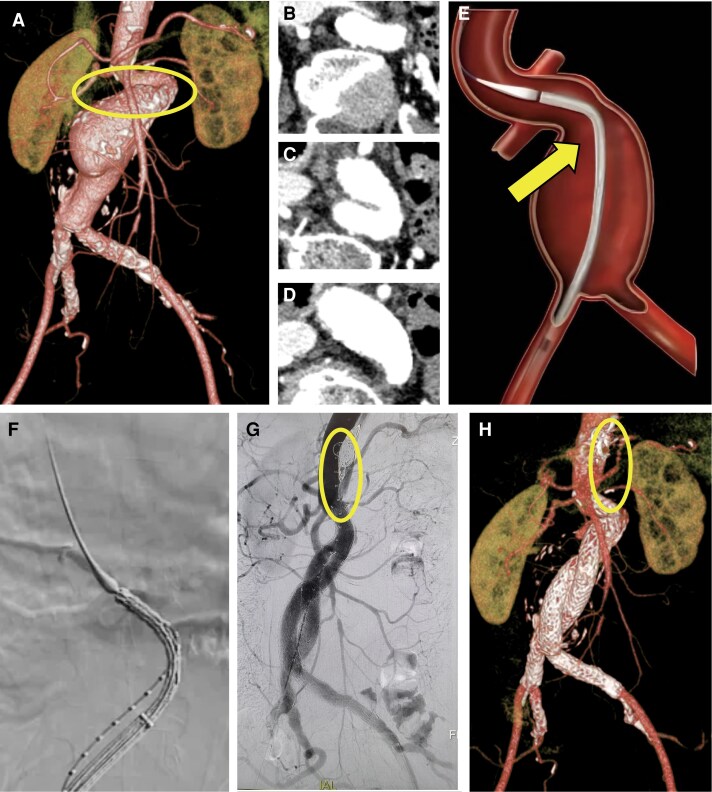
Preoperative imaging, procedural illustration, and postoperative outcome of endovascular repair using the wall–push technique. (*A*) Three–dimensional reconstruction of the preoperative computed tomography angiography (demonstrating an infrarenal abdominal aortic aneurysm with a severely angulated and tapered neck. (*B–D*) Representative axial computed tomography angiography slices showing the tortuous infrarenal neck and marked calcification of the bilateral iliac arteries. (*E*) Schematic diagram illustrating the wall–push technique, in which the stent–graft delivery system is gently pushed against the outer curvature of the vessel wall to facilitate alignment during deployment. The arrow indicates the direction of the gentle pressure applied against the outer curvature. (*F*) Intraoperative fluoroscopic view showing application of the wall–push technique in the angulated aneurysm neck. (*G*) Angiogram demonstrating an iatrogenic abdominal aortic dissection above the left renal artery caused by inadvertent forward displacement of the guidewire during the manoeuvre. (*H*) Postoperative 3D computed tomography angiography reconstruction confirming satisfactory stent–graft position and exclusion of the aneurysm, with restoration of antegrade flow.

An 80-year-old female with a 10-year history of conservatively managed AAA presented with persistent abdominal pain for over two months. Her medical history included type 2 diabetes mellitus and carotid artery atherosclerotic plaque. She had no history of hypertension, smoking, or coronary artery disease.

Computed tomography angiography (CTA) with three-dimensional reconstruction revealed: Maximum aneurysm diameter of 5.8 cm × 5.5 cm; infrarenal neck length of 18 mm; neck diameter of 21 mm (proximal) tapering to 19 mm (distal); suprarenal angulation of 45°; infrarenal neck angulation of 75°; and bilateral iliac arteries with moderate to severe calcification (*Table 2*).

**Table 2:** Preoperative CTA anatomical measurements

Parameter	Measurement	IFU threshold^a^
**Aneurysm dimensions**		
Maximum diameter (anteroposterior)	5.8 cm	—
Maximum diameter (transverse)	5.5 cm	—
**Infrarenal neck**		
Length	18 mm	≥15 mm
Proximal diameter	21 mm	19–29 mm
Distal diameter	19 mm	—
**Angulation**		
Suprarenal angulation	45°	≤60°
Infrarenal neck angulation	75°	≤60°^b^
Aortic bifurcation angle	65°	—
**Iliac arteries**		
Right common iliac artery diameter	14 mm	—
Right common iliac artery calcification	Moderate	—
Left common iliac artery diameter	16 mm	—
Left common iliac artery calcification	Severe, circumferential	—
Bilateral external iliac artery diameter	8 mm	≥7 mm

^a^IFU, Instructions for Use for the GORE® EXCLUDER® C3 device.

^b^Patient exceeded the recommended threshold, classifying this as an outside-IFU deployment.

CTA, computed tomography angiography; EVAR, endovascular aneurysm repair.

EVAR using the GORE® EXCLUDER® C3 system was planned. Given the neck angulation exceeded the recommended threshold of ≤60°, this was classified as an outside-IFU (the Instructions for Use) deployment.

During the procedure, the ‘wall-push technique’ was employed to navigate the challenging angulation. This technique involves positioning a stiff guidewire in the ascending aorta, then applying gentle forward pressure to the delivery system against the outer vessel wall, creating a fulcrum effect to overcome acute angulation and achieve coaxial alignment.

The GORE® EXCLUDER® C3 stent-graft (23–14 mm × 120 mm) was advanced over an Amplatz stiff guidewire. However, during execution of the wall-push technique, inadequate guidewire control resulted in inadvertent forward displacement, causing an iatrogenic dissection above the left renal artery.

The dissection was promptly managed with coil embolization of the false lumen using five Interlock coils. The procedure then proceeded to definitive aneurysm exclusion. The main body was deployed just distal to the renal arteries, followed by bilateral iliac limb extensions positioned at the internal iliac artery origins. Balloon moulding was performed at all seal zones.

Final angiography demonstrated a small type II endoleak from a lumbar artery. Given its low-flow characteristics, conservative management with surveillance imaging was elected.

The patient was discharged on postoperative Day 4. Discharge medications included continuation of aspirin 100 mg daily, atorvastatin 20 mg daily, and amlodipine 5 mg daily. Clopidogrel 75 mg daily was added for 3 months given the coil embolization of the false lumen. At 1-month follow-up, she remained asymptomatic with stable aneurysm sac diameter, complete thrombosis of the embolized false lumen, and patent renal arteries. The surveillance protocol includes CTA at 6 and 12 months.

## Discussion

EVAR has emerged as the preferred treatment modality for AAA in elderly patients, offering reduced perioperative mortality and morbidity compared to open surgical repair.^1–3^

The GORE® EXCLUDER® C3 system offers enhanced flexibility for navigation through tortuous anatomy. In our case, the severely angulated infrarenal neck necessitated the wall-push technique to achieve adequate positioning.^4^

It should be noted that the wall-push technique has been more extensively described in the thoracic endovascular aortic repair literature, where it is employed to navigate the aortic arch and manage challenging landing zones.^4,5^ However, the underlying principles—utilizing controlled wall contact to create a fulcrum effect for device advancement through severe angulation—are directly transferable to complex infrarenal EVAR cases with hostile neck anatomy. Georgiadis *et al*. have summarized various tips and techniques for managing challenging EVAR anatomy, emphasizing the importance of operator experience and technical adaptability when facing anatomical constraints.^6^

The decision to proceed with standard EVAR outside IFU (the Instructions for Use) remains controversial. Recent meta-analyses demonstrate acceptable mid-term outcomes, although with higher rates of Type Ia endoleak and reintervention compared to within-IFU cases.^7,8^ In our case, given the patient's advanced age and comorbidities, standard EVAR with adjunctive techniques was preferred over more complex alternatives such as fenestrated EVAR. However, these approaches introduce additional complexity and potential complications that may not be justified in all patients.^9^

Achieving adequate seal in severely angulated necks requires attention to several technical factors. Coaxial alignment between the stent-graft and the aortic wall at the proximal landing zone is critical to ensure circumferential apposition and minimize the risk of Type Ia endoleak. The wall-push technique specifically aims to achieve this coaxial orientation prior to deployment. However, even with optimal positioning, the acute angulation may result in incomplete expansion of the proximal covered stent, creating a ‘bird-beak’ configuration that predisposes to endoleak and may compromise long-term seal durability.^10^

Distal seal is equally important, particularly in cases with aneurysmal or ectatic iliac arteries. In our case, the iliac extensions were deployed with their distal ends positioned at the origins of the internal iliac arteries to maximize landing zone length while preserving pelvic perfusion. Balloon moulding of all seal zones was performed to optimize stent-graft apposition.

Endoleaks in challenging anatomy occur more frequently than in straightforward cases. Recent registry data suggest that Type Ia endoleak rates may be 2–3 times higher in patients with hostile neck anatomy treated outside IFU.^11,12^ This underscores the importance of rigorous surveillance protocols with low thresholds for secondary intervention. The identification of a Type II endoleak in our case, while managed conservatively, requires vigilant follow-up given the underlying anatomical complexity.

Potential pitfalls of the wall-push technique include (1) excessive forward pressure causing guidewire advancement and vessel injury, as occurred in our case; (2) plaque disruption in heavily calcified aortas; (3) incomplete stent-graft expansion due to residual angulation; and (4) Type Ia endoleak due to suboptimal seal zone apposition.^13^

The iatrogenic dissection in our case resulted from inadvertent guidewire advancement during the wall-push manoeuvre. Potential pitfalls of this technique include: (1) excessive forward pressure causing guidewire advancement and vessel injury; (2) plaque disruption in calcified aortas; (3) incomplete stent-graft expansion; and (4) Type Ia endoleak due to suboptimal apposition.^14^

Several technical considerations deserve emphasis when using the C3 delivery system in complex anatomy. First, proper guidewire selection is crucial—while stiff wires provide better support, they also carry higher risk of vessel injury.^15^ When employing the wall-push technique, the assistant controlling the guidewire must maintain constant vigilance to prevent inadvertent advancement as the operator applies forward pressure to the delivery system. Second, the enhanced flexibility of the C3 system, while advantageous for navigation, may require more careful control during deployment to prevent unintended movement. Third, team coordination is essential, with clear communication between the primary operator and assistant managing the guidewire.

Comprehensive preoperative planning is essential. Three-dimensional CTA reconstruction helps identify optimal projection angles and predict potential challenges. Accurate measurement of neck angulation determines whether the wall-push technique is necessary and anticipates the degree of force required.

Long-term surveillance following EVAR with iatrogenic dissection requires monitoring for dissection progression, false lumen thrombosis, and branch vessel compromise, in addition to standard endoleak surveillance.^16^

## Conclusion

This case demonstrates successful treatment of a complex AAA using the GORE® EXCLUDER® C3 system with the wall-push technique. The iatrogenic dissection encountered was effectively managed with coil embolization. This case highlights both the utility and potential risks of advanced EVAR techniques, emphasizing the need for meticulous guidewire control, comprehensive preoperative planning, and preparedness for complication management.

## Data Availability

The data underlying this case report are available within the article. Additional data that support the findings of this study are available from the corresponding author upon reasonable request, subject to patient privacy considerations.

## Lead author biography

I am an 8-year programme medical student at Shanghai Medical College, Fudan University, with a research interest in vascular surgery, particularly in the diagnosis, management, and pathophysiological mechanisms of aortic dissection and abdominal aortic aneurysm.

## Notes

**Consent:** The authors confirm that written consent for submission and publication of this case report including images and associated text has been obtained from the patient in line with COPE guidance.
